# IPSC-NSCs-derived exosomal let-7b-5p improves motor function after spinal cord Injury by modulating microglial/macrophage pyroptosis

**DOI:** 10.1186/s12951-024-02697-w

**Published:** 2024-07-09

**Authors:** Jie Liu, Guang Kong, Chenlin Lu, Juan Wang, Wenbo Li, Zhengming Lv, Jian Tong, Yuan Liu, Wu Xiong, Haijun Li, Jin Fan

**Affiliations:** 1 Department of Orthopaedics, Taizhou School of Clinical Medicine, The Affiliated Taizhou People’s Hospital of Nanjing Medical University, Nanjing Medical University, 366 Taihu Road, Taizhou, Jiangsu China; 2 Department of Orthopaedics, The First Affiliated Hospital of Nanjing Medical University, 300 Guangzhou Road, Nanjing, Jiangsu China; 3 Department of Orthopaedics, Xijing Hospital, Fourth Military Medical University, Xi’an, Shaanxi China; 4 Department of Clinical Research Center, Taizhou School of Clinical Medicine, The Affiliated Taizhou People’s Hospital of Nanjing Medical University, Nanjing Medical University, 366 Taihu Road, Taizhou, Jiangsu China; 5 Department of human anatomy, School of Basic Medicine, Nanjing Medical University, Nanjing, Jiangsu China; 6 Songjiang Institute, Shanghai Jiao Tong University School of Medicine, Shanghai, China

**Keywords:** Induced pluripotent stem cell-derived neural stem cells, Exosomes, Spinal cord injury, Pyroptosis, Neuroinflammation

## Abstract

**Background:**

Following spinal cord injury (SCI), the inflammatory storm initiated by microglia/macrophages poses a significant impediment to the recovery process. Exosomes play a crucial role in the transport of miRNAs, facilitating essential cellular communication through the transfer of genetic material. However, the miRNAs from iPSC-NSCs-Exos and their potential mechanisms leading to repair after SCI remain unclear. This study aims to explore the role of iPSC-NSCs-Exos in microglia/macrophage pyroptosis and reveal their potential mechanisms.

**Methods:**

iPSC-NSCs-Exos were characterized and identified using transmission electron microscopy (TEM), nanoparticle tracking analysis (NTA), and Western blot. A mouse SCI model and a series of in vivo and in vitro experiments were conducted to investigate the therapeutic effects of iPSC-NSCs-Exos. Subsequently, miRNA microarray analysis and rescue experiments were performed to confirm the role of miRNAs in iPSC-NSCs-Exos in SCI. Mechanistic studies were carried out using Western blot, luciferase activity assays, and RNA-ChIP.

**Results:**

Our findings revealed that iPSC-NSCs-derived exosomes inhibited microglia/macrophage pyroptosis at 7 days post-SCI, maintaining myelin integrity and promoting axonal growth, ultimately improving mice motor function. The miRNA microarray showed let-7b-5p to be highly enriched in iPSC-NSCs-Exos, and LRIG3 was identified as the target gene of let-7b-5p. Through a series of rescue experiments, we uncovered the connection between iPSC-NSCs and microglia/macrophages, revealing a novel target for treating SCI.

**Conclusion:**

In conclusion, we discovered that iPSC-NSCs-derived exosomes can package and deliver let-7b-5p, regulating the expression of LRIG3 to ameliorate microglia/macrophage pyroptosis and enhance motor function in mice after SCI. This highlights the potential of combined therapy with iPSC-NSCs-Exos and let-7b-5p in promoting functional recovery and limiting inflammation following SCI.

**Supplementary Information:**

The online version contains supplementary material available at 10.1186/s12951-024-02697-w.

## Introduction

Spinal cord injury (SCI) is predominantly attributed to traumatic events, including traffic accidents, falls from heights, and sports-related injuries. SCI often results in debilitating motor, sensory, and autonomic dysfunction, contributing to a high prevalence of disability and mortality [1, 2]. This condition inflicts profound physical and psychological trauma upon affected individuals and imposes substantial economic burdens on nations. SCI incidence is rapidly rising worldwide, but a definitive cure remains elusive [3].

SCI is categorized into primary and secondary forms, with secondary injuries resulting in permanent structural damage to the spinal cord [4]. Therefore, mitigating the harm caused by secondary injuries is of paramount importance. Microglia—often regarded as tissue-resident macrophages—play pivotal roles in maintaining the spinal cord microenvironment within the central nervous system [5]. Their responsibilities include clearing apoptotic cells and aberrant neural connections to uphold spinal cord homeostasis [6, 7]. However, following SCI, microglia/macrophages aggregate swiftly within the injured region and become highly active. These activated microglial/macrophages release reactive oxygen species and cytokines, leading to a secondary neuroinflammatory injury [8]. Studies have suggested that suppressing the abnormal neuroinflammatory cascades post-SCI can improve motor function recovery [9, 10].

Pyroptosis—a recently discovered form of cell death with pro-inflammatory and lytic properties—activates inflammasomes, such as NLRP3, during cellular inflammation [11]. These activated inflammasomes cleave Caspase-1 precursors, promoting GSDMD cleavage and isolation. Consequently, GSDMD-N-terminal fragments translocate to the cell membrane, forming pores that induce cell swelling, rupture, and release pro-inflammatory cytokines, including IL-1β, amplifying the inflammatory response through pyroptosis [12, 13]. Studies have hinted at the potential involvement of microglial/macrophage pyroptosis in the neuroinflammatory process, and inhibiting this process could alleviate post-SCI neuroinflammation levels and neuronal apoptosis [14, 15]. Nevertheless, the precise molecular mechanisms underlying microglial/macrophage pyroptosis post-SCI remain unclear.

Neural stem cells (NSCs) play a pivotal role in SCI repair by exerting anti-inflammatory and immune-regulatory effects, participating in remyelination and axonal regeneration, and releasing trophic factors that support and enhance the growth of injured neurons [16, 17]. However, sourcing NSCs is limited, and their survival and integration at the injury site are challenging [18]. These aforementioned issues are addressed by the emergence of induced pluripotent stem cells (iPSCs) [19]. Shiya Yamanaka first reported the induction of iPSCs in 2006 [20], which have diverse applications in drug screening, cell replacement therapies, treating cardiovascular diseases and neurological [21–23]. Induced pluripotent stem cell-derived neural stem cells (iPSC-NSCs) have shown promise in neurological disorders like Alzheimer’s and multiple sclerosis [24]. However, their role in pyroptosis post-SCI remains underexplored. The present study investigated the potential contributions of iPSC-NSCs and their exosomes in microglial/macrophage pyroptosis and subsequent neuroinflammatory responses post-SCI.

Exosomes contain miRNAs, proteins, nucleic acids, and other small molecules, among which miRNAs are short RNA molecules that can regulate the post-transcriptional silencing of target genes. A single miRNA can target hundreds of mRNAs and affect the expression of genes with interacting functions, playing an important role in regulating cell function [25, 26]. Exosomes can act as a protective shell to limit the rapid degradation of miRNA in an enzymic environment, effectively deliver miRNA to a designated location, and maintain its stability [27]. Exosomes and miRNAs have been extensively studied in the treatment of spinal cord injury. For example, it has been proven that exosomes derived from umbilical cord mesenchymal stem cells repair spinal cord injury through the inclusion of miR-29b-3p [28], while exosomes derived from bone marrow mesenchymal stem cells can use miR-216a-5p to change the polarization direction of microglia [8].

However, whether iPSC-NSCs derived exosomes and their miRNAs have an effect on focal death after SCI remains unclear. As a miRNA, we hypothesized that let-7b-5p mediated the mechanism of inflammation improvement and conducted experimental studies.

Our findings demonstrate that iPSC-NSCs transplantation reduces microglial/macrophage pyroptosis and enhances motor function post-SCI. Additionally, we found that let-7b-5p overexpression in iPSC-NSCs-derived exosomes significantly reduces post-SCI inflammation and improves motor function. These insights offer valuable strategies for treating SCI and related neuroinflammatory disorders.

## Materials and methods

### BV2 microglial cell culture

The Shanghai Cell Research Center (Shanghai, China) provided the mouse BV2 microglial cell line. These cells were cultivated in DMEM medium supplemented with 1% penicillin/streptomycin and 10% fetal bovine serum. They were also kept in an environment with 5% CO_2_ at 37 °C. After reaching approximately 80% confluence, the cells were dissociated using trypsin and passaged for further experimental use. Pyroptosis induction experiments on the BV2 cells were performed using LPS and ATP.

### Culture of induced pluripotent stem cell-derived neural stem cells and adoptive transfer

The well-validated iPSC-NSCs cell line was obtained from Cellapy Biotechnology Co. (Cat# CA2301106, Beijing, China). iPSC-NSCs were cultured in NeuroEasy recovery and maintaining medium (Cellapy Biotechnology Co. Beijing, China) under 5% CO_2_ at 37 °C, according to the manufacturer’s guidelines.

The cells were cultured on coverslips and then fixed in a 4% paraformaldehyde solution. Following fixation, tpermeabilization was carried out using 0.05% Triton X-100, followed by blocking with 5% BSA. After blocking, the cells were exposed to primary antibodies (Nestin, SOX2, PAX6, and OCT4) at 4 ℃ overnight. Subsequently, the cells underwent treatment the next day with matching secondary antibodies and DAPI. The resulting staining patterns were visualized using a fluorescence microscope. Immediately after spinal cord injury, recipient mice received intrathecal injections of purified fresh iPSC-NSCs (3 × 10^6^). Control mice received the same amount of phosphate buffered saline (PBS).

### Isolation, characterization, and injection of induced pluripotent stem cell-derived exosomes

The culture supernatant of iPSC-NSCs was harvested for exosomes. After extracting the supernatant, exosomes were isolated using a differential centrifugation method. This method involved centrifuging the cell debris for 10 min at 2000 g, followed by a 10,000 g centrifugation step for 10 min to eliminate apoptotic bodies. Finally, a 100,000 g centrifugation for 90 min was conducted to separate the supernatant and collect exosomes potentially containing impurities. The collected exosomes were subsequently resuspended in 20 mL of phosphate-buffered saline (PBS) and subjected to an additional centrifugation at 100,000 g for 90 min. The exosomes were then resuspended in 50 mL of PBS and kept for further applications at -80 °C.

To ascertain the presence of extracted exosomes, we used TEM and NTA to examine exosomal morphology, diameter distribution, and abundance. Additionally, exosomal surface markers (Alix, TSG101, CD9, CD63, and CD81) were detected using western blotting. Following SCI, the mice were administered intrathecal injections of iPSC-NSCs-derived exosomes at 20 µg/µL. In comparison, the mice in the control group received a similar volume of PBS.

### Exosome uptake by microglia

The exosomes were labeled with fluorescent markers following the manufacturer’s guidelines. Exosomes suspended in PBS were subjected to a 4 mg/mL Dil solution (Molecular Probes, Oregon, USA) and cultured. The surplus fluorescent dye was removed by centrifuging the mixture at 100,000 g for 1 h at 4 °C. Following a 24 h co-culture with Dil-labeled exosomes, microglia were subjected to a PBS wash and then fixed with a 4% paraformaldehyde solution. Finally, laser confocal microscopy was used for observing the uptake of Dil-labeled exosomes by microglia.

### Animals

The Nanjing Medical University Ethics Committee approved the animal experiments for this study. The Laboratory Animal Center of Nanjing Medical University (Nanjing, China) supplied eight-week-old C57BL/6 mice, kept them in SPF conditions, and provided sufficient food and water.

### Spinal cord injury model

A spinal cord injury (SCI) animal model was established using mice aged between 8 and 10 weeks. The mice were initially sedated using isoflurane inhalation, and then the spinal cord became visible following a laminectomy at the T8 vertebra. Afterward, an impactor was used to strike the spinal cord with a 5 g rod that had been dropped from a height of 6.5 cm, causing SCI. Immediate post-SCI procedures involved suturing the muscles and closing the skin. Bladder emptying was performed manually thrice daily until the bladder function was re-reflexively regulated.

#### Basso mouse scale (BMS) behavioral analysis

The quantification of various parameters, including hind limb range of motion, trunk position, stability, paw position, toe spacing, tail position and coordination between front and back limbs, were conducted at pre-SCI and specific intervals of 1, 3, 7, 14, 21, and 28 days post-SCI. Motor function was evaluated using the Basso Mouse Scale (BMS) score, with measures taken to ensure the observer’s blinding to group assignments throughout the observation period.

#### Footprint analysis

To evaluate the restoration of motor function, we conducted a mouse footprint analysis at 28 days post-SCI. For this approach, the mice were first given blue dye on their front and red dye on their hind paws. The mice were made to run in a straight line on a predetermined piece of test paper. Measurements and analyses of stride lengths and widths were conducted by two impartial examiners who were not aware of the treatment program .

### Swimming test

Swimming assessments were conducted on mice at pre- and post-SCI. During these assessments, mice were placed within a tank and prompted to traverse from one extremity to another. Subsequently, swimming postures were documented, and the evaluation of motor function recovery was conducted utilizing the Louisville Swim Scale.

### Electrophysiology testing

Motor-evoked potentials (MEPs) in mice were evaluated using electromyography at 28 days post-SCI. The stimulating electrode was surgically implanted on the rostral aspect of the spinal cord, whereas the recording electrode was positioned at the periphery of the quadriceps femoris muscle. A reference electrode was placed into the distal tendon of the hind limb muscles, whereas a ground electrode was positioned subcutaneously. Stimulation was delivered using a single square-wave pulse with the following parameters: 10 mA, 0.5 ms, and 1 Hz.

### Western blot assay

Proteins were extracted from target cells or tissues by lysing them with RIPA lysis buffer. Subsequently, a BCA test was used to determine the protein concentrations. Afterward, an equal amount of proteins underwent separation on SDS-PAGE gels, were transferred onto PVDF membranes, and subsequently blocked using a BSA solution for 1 h at room temperature. Following this, the membranes were exposed to primary antibodies overnight at 4 °C. Afterwards, the membranes were washed in TBST and incubated for 2 h at room temperature with secondary antibodies. The membrane underwent three TBST washes, and the protein bands were identified using an ECL reagent and the Bio-Rad ChemiDoc XRS + Gel Imaging System. Image J was used to assess gray values. The following antibodies were used by us: anti-NLRP3 (1:1000), anti-GSDMD (1:1000), anti-GSDMD-N (1:1000), anti-Caspase-1 (1:1000), anti-P20 (1:1000), anti-IL-1β (1:1000), anti-Alix (1:1000), anti-TSG101 (1:1000), anti-CD9 (1:1000), anti-CD63 (1:1000), anti-CD81 (1:1000), anti-LRIG3 (1:2000), and anti-GAPDH (1:50000).

### Immunofluorescence staining assays

The injured spinal cords were extracted and fixed overnight in a 4% paraformaldehyde solution after perfusing the mouse hearts first with 0.9% saline and then with 4% paraformaldehyde. Following dehydration, the samples were sectioned into 10-µm-thick slices. Before culturing, the slices were treated with a 10% BSA blocking solution and then incubated at 4 °C overnight with primary antibodies (anti-IBA1, anti-GSDMD, anti-Caspase-1, anti-NF200 and anti-NeuN). The slices were then incubated for 2 h at room temperature with secondary antibodies .

The cells were initially permeabilized with 0.05% Triton X-100 after being fixed for 30 min in 4% paraformaldehyde. Subsequently, they were blocked with 5% BSA to enhance the effectiveness of cell immunofluorescence staining. Later, the cells were incubated with primary antibodies (anti-IBA1, anti-Caspase-1 and anti-GSDMD) for an overnight period at 4 °C before undergoing treatment with secondary antibodies. Following three PBS washes, the nuclei were eventually counterstained using DAPI.

### Luxol fast blue staining

To assess the quantity of neurons and the integrity of the neuronal myelin sheath post-SCI, we used Luxol Fast Blue Staining Reagent (Servicebio) according to the manufacturer’s instructions. The stained sections were examined under a Leica optical microscope.

### miRNA microarray assay and Vector constructs

Hangzhou Kaitai Biotech Co., Ltd. (Hangzhou, China) performed miRNA array experiments on iPSC-NSCs and iPSC-NSCs-Exos. Each experimental set comprised three samples. Sequencing libraries were generated using NEBNext^R^ Ultra™ small RNA Sample Library Prep Kit for Illumina^R^(NEB, USA) following manufacturer’s recommendations and index codes were added to attribute sequences to each sample. Quality assurance of these libraries was done via an Agilent 2100 Bioanalyzer (Agilent Technologies, CA, USA). Subsequently, using the TruSeq PE Cluster Kitv3-cBot-HS on a cBot Cluster Generation System, the indexed samples were clustered. Finally, sequencing was executed on an Illumina platform, generating the desired reads. Lentiviral vector (GenePharma, Shanghai, China) was used to construct the LV2-let-7b-5p mimic vector (LV2-let-7b-5p) and the LV2-let-7b-5p inhibitor vector (ANTI-let-7b-5p). Negative controls were constructed with LV2 empty lentiviruses (LV2-NC and ANTI-NC).

### Target gene prediction and dual-luciferase reporter gene assay

The let-7b-5p target genes were predicted using the TargetScan and miRDB databases. The sequence corresponding to the 3’-UTR of LRIG3 mRNA, which includes the wild-type (WT) or mutant (MUT) let-7b-5p binding site, was generated by Hippo Biotechnology Co., Ltd. (Nanjing, China). The let-7b-5p sequences were cloned into the pGL3 luciferase reporter vector (Promega, Madison, WI, USA) at XbaI and FseI restriction sites. Subsequently, the engineered pGL3 luciferase reporter vector was introduced into HEK293T cells. After transfection, the fluorescence microscopy was utilized to observe the expression of genes labeled with fluorescent markers in HEK293T cells for 24 h. The Dual-Luciferase Reporter Assay System kit was used to measure the luciferase expression.

### Isolation of RISC-associated RNA

Following 1% formaldehyde fixation, chromatin fragmentation was performed on BV2 microglia cells that were overexpressing let-7b-5p or miR-NC. Subsequently, cellular lysis in NETN buffer was performed, and immunoprecipitation was carried out by incubating the lysate with Dynabeads Protein A (Invitrogen), supplemented with either IgG control, anti-Pan-Ago, or clone 2A8 antibody (Millipore). The RNA, isolated through proteinase K digestion from immunoprecipitation, was extracted utilizing the phenol/chloroform/isopropanol method. Further purification involved RNA isolation through glycogen ethanol precipitation, followed by treatment with DNase I.

### RNA extraction and quantitative RT-PCR assay

With the use of the TRIzol reagent (Invitrogen, Carlsbad, CA, USA), total RNA from cells and exosomes was extracted. The cDNA for miRNA was produced using the Hairpin-itTM miRNA qPCR Quantitation Kit (GenePharma, China), and the cDNA for mRNA was produced using the PrimeScript RT reagent Kit (Takara, Japan). Subsequently, utilizing the TB Green^®^ Premix Ex TaqTM Kit (Takara, Japan), the qRT-PCR experiment was performed. The 2^−ΔΔCT^ method was utilized to normalize the mRNA and miRNA expression levels to GAPDH and U6, respectively, in order to calculate the relative expression.

### miRNA microarray data process

Quality control: Raw data (raw reads) of fastq format were firstly processed through in-house perl scripts. In this step, clean data(clean reads) were obtained by removing reads containing adapter, reads containing ploy-N and low quality reads from raw data. And reads were trimmed and cleaned by removing the sequences smaller than 15nt or longer than 35 nt. At the same time, Q20, Q30, GC-content of the clean datawere calculated. All the downstream analyses were based on clean data with high quality.

Quantification of miRNA expression levels: miRNA expression levels were estimated for each sample: sRNA were mapped back onto the precursor sequence. Readcount for each miRNA was obtained from the mapping results.

Differential expression analysis: Differential expression analysis of two conditions/groups was performed using the DESeq R package. DESeq provide statistical routines for determining differential expressionin digital gene expression data using a model based on the negative binomial distribution. The resulting Pvalues were adjusted using theBenjamini and Hochberg’s approach for controlling the false discovery rate. Genes with an adjusted P-value < 0.01 and absolute value of log2(Fold change) > 1 found by DESeq were assigned as differentially expressed.

### Statistical assay

The data, which were gathered from more than three separate experiments, are displayed as the mean ± standard deviation (mean ± SD). GraphPad Prism 8.0 was used for statistical analysis. We evaluated significant distinctions using Student’s t-test or one-way or two-way ANOVA, and significance was denoted by a p-value less than 0.05 (*p < 0.05; **p < 0.01; ***p < 0.001).

## Results

### Pyroptosis occurs in microglial/macrophages after spinal cord injury

**Fig. 1:**
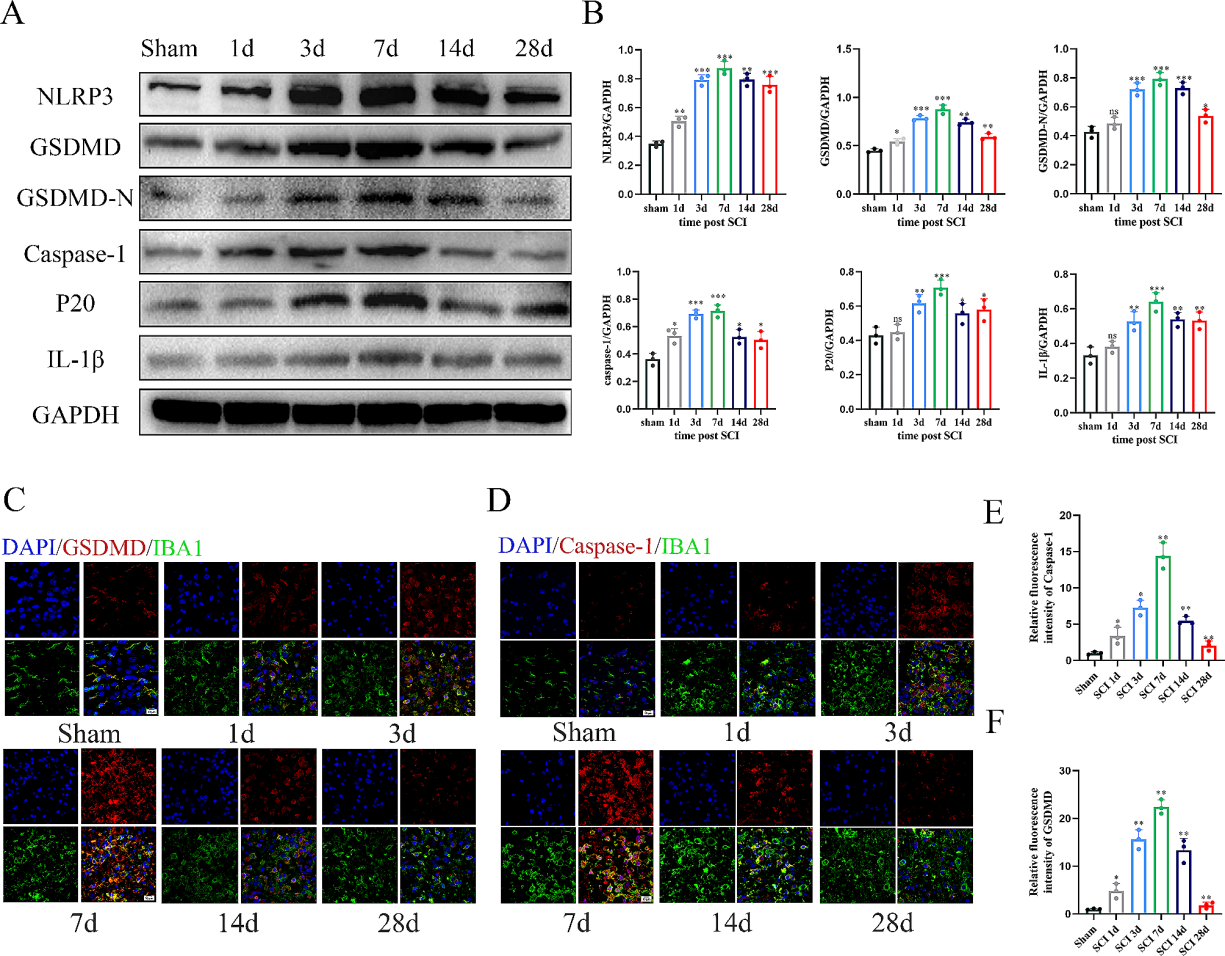
Pyroptosis occurs in microglial/macrophages after spinal cord injury. **(A)** Western blot analysis of pyroptosis-related protein expression at days 1, 3, 7, 14, and 28 following SCI; **(B)** Quantitative analysis of pyroptosis-related protein; **(C-D)** Representative Immunofluorescence images of GSDMD/Caspase-1 (red) and IBA-1 (green) in the SCI area after injury; **(E-F)** Quantifcation of fuorescence intensity of GSDMD and Caspase-1 aftert injury. *P < 0.05; **P < 0.01; ***P < 0.001 (*n* = 3 per group)

Our previous study found that the expression of pyroptosis related protein reached its peak 7 days after spinal cord injury and was expressed in microglia, astrocytes, neurons and other cells, but it mainly occurred in microglia/macrophages in the spinal cord region [14]. In the present study, we conducted a comprehensive series of experiments to further investigate the spatiotemporal characteristics of pyroptosis post-SCI. First, we used western blotting to detect the expression of key proteins involved in the pyroptotic pathway at different time points post-SCI. The expression levels of NLRP3, GSDMD, pro-Caspase-1, p20 and IL-1β significantly increased at 1 day post-SCI, with peak expression at 7 days post-SCI (Fig. 1A-B). Subsequently, we performed immunofluorescent staining to assess the co-localization of pyroptosis markers (GSDMD and Caspase-1) with the microglia/macrophages marker IBA1 (Fig. 1C–F). Consistent with the western blotting findings, our results demonstrated that pyroptosis was most pronounced in microglia/macrophages at 7 days post-SCI.

### Increased iPSC-NSCs infiltration in the spinal cord attenuates microglial/macrophage pyroptosis in vivo

**Fig. 2:**
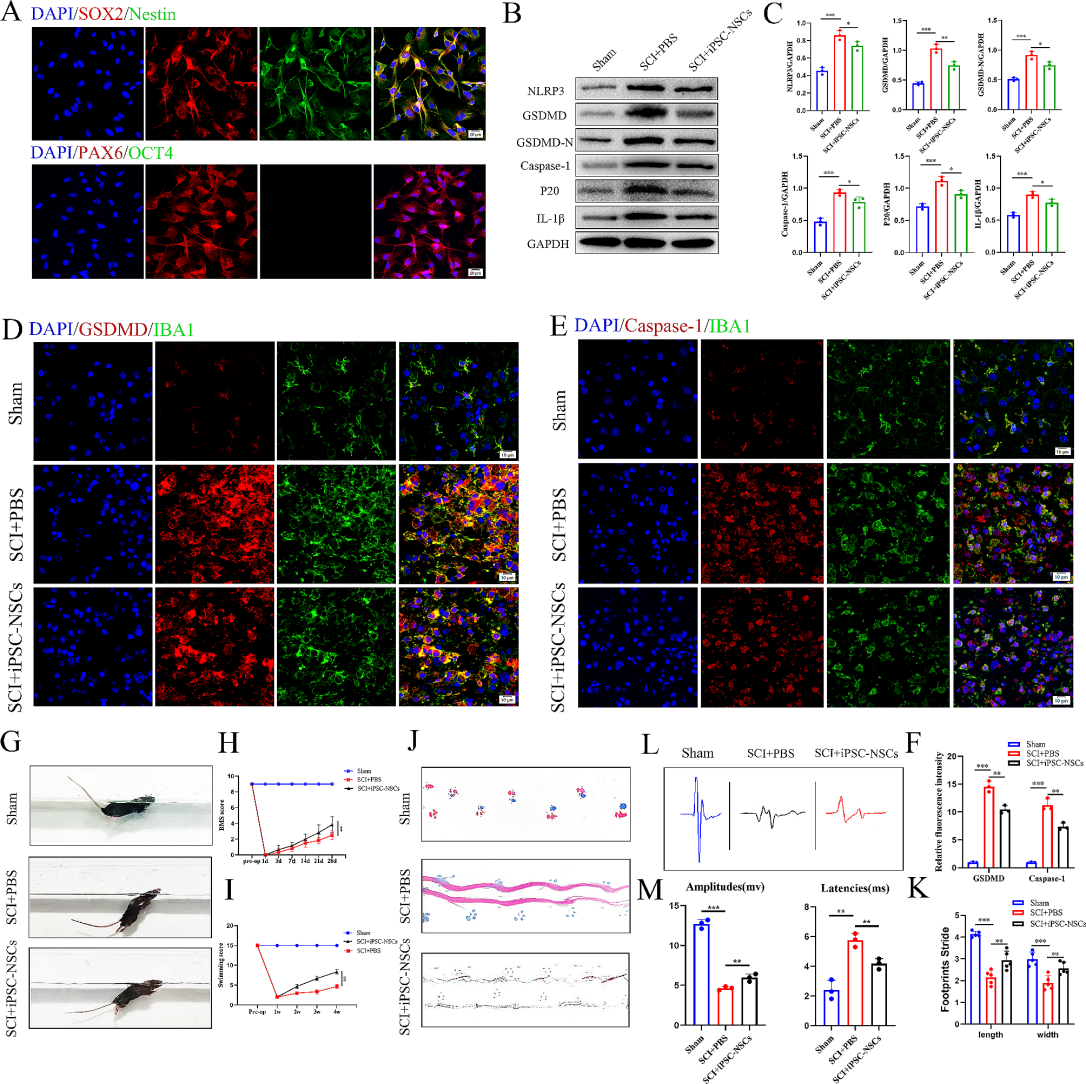
Increased iPSC-NSCs infiltration in the spinal cord attenuates microglial/macrophage pyroptosis and promotes functional recovery after SCI. **(A)** Immunofluorescence staining showing expression of the iPSC-NSCs markers Nestin, SOX2 and PAX6; **(B)** Western blot analysis of pyroptosis-related protein expression at day 7 after injury in sham group, SCI + PBS, and SCI + iPSC-NSCs mice; **(C)** Quantitative analysis of pyroptosis-related protein; **(D-E)** Representative immunofluorescence images of GSDMD/Caspase-1 (red) and IBA-1 (green) in sham group and SCI mice treated with PBS and iPSC-NSCs; **(F)** Quantification of fuorescence intensity of GSDMD and Caspase-1 at sham group and day 7 after injury; **(G-H)** Typical diagrams of swimming tests in sham group, SCI + PBS, and SCI + iPSC-NSCs mice at day 28 after injury; **(I)** BMS scores of sham group, treated with PBS and iPSC-NSCs mice during the recovery period 28 days after injury (*n* = 3 per group); **(J-K)** Typical diagrams of footprint tests in sham group, SCI + PBS, and SCI + iPSC-NSCs mice at day 28 after injury (*n* = 5 per group); **(L-M)** Electrophysiological assessment with MEP analysis in in sham group, SCI + PBS, and SCI + iPSC-NSCs mice at day 28 post-injury. *P < 0.05; **P < 0.01; ***P < 0.001 (*n* = 3 per group)

We verified whether the cells were iPSC-NSCs cells by observing the expression of Nestin, SOX2, and PAX6 (Fig. 2A). Subsequently, to evaluate the impact of iPSC-NSCs on microglial/macrophage pyroptosis post-SCI, we administered iPSC-NSCs transplantation at the site of injury in mice.We transfected the green fluorescent protein expression plasmid into iPSC-NSCs to produce EGFP labeled cells, and then injected the EGFP labeled cells into the injured site of mice. Small animal imaging systems were used to observe cell viability and migration of EGFP labeled cells after transplantation in vivo. Representative IVIS images show that cells can survive and infiltrate at the site of spinal cord injury (Figs. S1A-B). Western blotting was employed to evaluate the levels of NLRP3, GSDMD, GSDMD-N, Caspase-1, p20 and IL-1β in the injury epicenter at 7 days post-SCI. Our results indicated a significant reduction in the expression levels of these proteins in the iPSC-NSCs mice compared with the PBS mice (Fig. 2B-C). Furthermore, we conducted immunofluorescence analysis of the injury site to reveal that iPSC-NSCs mice exhibited a lower presence of IBA1^+^/GSDMD^+^ and IBA1^+^/Caspase-1^+^ compared with the PBS mice. (Figs. 2D-F). During the 28-day recovery process post-SCI, we conducted multiple behavioral assessments on mice. Swimming tests demonstrated that the iPSC-NSCs mice exhibited improved gait and enhanced motor coordination (Fig. 2G-H). Consistent with the results of the swimming tests, BMS scores and footprint analysis also indicated superior motor recovery in iPSC-NSCs mice (Fig. 2I-K). The electrophysiologic test demonstrated that the iPSC-NSCs mice exhibited increased amplitudes and shorter MEP latency than the PBS mice (Fig. 2L-M). The above findings suggest that iPSC-NSCs transplantation can enhance functional recovery post-SCI while attenuating microglial/macrophage pyroptosis.

### iPSC-NSCs-derived exosomes attenuate pyroptosis in BV2 microglia in vitro

**Fig. 3:**
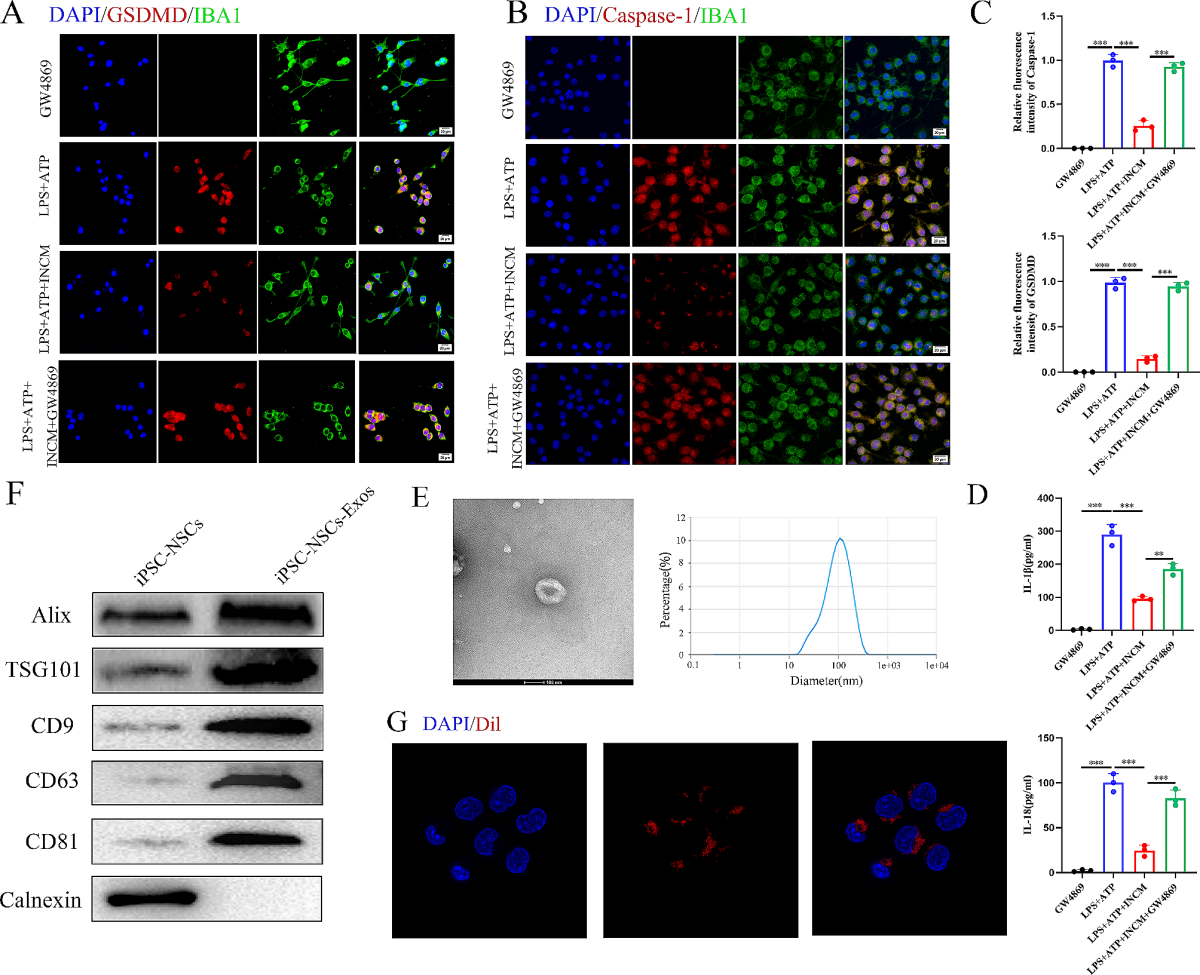
iPSC-NSCs-derived exosomes suppressed microglia pyroptosis. **(A-B)** Representative Immunofluorescence staining of GSDMD/Caspase-1(red) and IBA1(green) in the microglia; **(C)** Quantifcation of fuorescence intensity of GSDMD and Caspase-1 in the microglia; **(D)** ELISA analysis of IL-1β and IL-18 in BV2 cells; **(E)** Morphology of iPSC-NSCs-derived exosomes under TEM (50–150 nm) and NTA analysis of particle size distribution of exosomes; **(F)** Western blot analysis of the exosomes biomarkers Alix, TSG101, CD9, CD63, CD81 and negative control Calnexin; **(G)** The red fuorescence dye Dil-labeled exosomes was uptaken into microglia.*P < 0.05; **P < 0.01; ***P < 0.001 (*n* = 3 per group)

To further explore the mechanism that iPSC-NSCs mitigate microglial pyroptosis, we harvested iPSC-NSCs-conditioned medium (INCM). After co-culture with INCM, BV2 cells were treated with LPS (1 µg/mL) for 5.5 h, and then incubated with ATP (5 mM/ mL) for 0.5 h to induce pyroptosis models in vitro, microglia were subjected to immunofluorescent staining for IBA1, GSDMD, and Caspase-1 to evaluate pyroptosis. Compared with the LPS + ATP group, the LPS + ATP + INCM group displayed a substantial reduction in the expression levels of GSDMD and Caspase-1. Since exosomes play a critical role in intercellular communication and the transfer of genetic material, we speculated that iPSC-NSCs alleviate microglial pyroptosis by releasing exosomes. To test our speculation, we used the exosome inhibitor GW4869 (10 µM) to impede exosome secretion from iPSC-NSCs. This intervention reversed the reduced expression levels of GSDMD and Caspase-1 in the GW4869-pre-treated LPS + ATP + INCM group (Fig. 3A-C). ELISA analysis of IL-1β and IL-18 showed the same trend (Fig. 3D).

### iPSC-NSCs-derived exosomes contribute to the recovery of motor performance and alleviate pyroptosis post-SCI

To further explore the role of iPSC-NSCs-derived exosomes in the SCI microenvironment and their effect on microglial/macrophage pyroptosis, we extracted exosomes (iPSC-NSCs-Exos) from the INCM. Subsequently, transmission electron microscopy (TEM) revealed the presence of classic nanoparticles with diameters ranging from 50 to 150 nm, whereas nanoparticle tracking analysis (NTA) indicated a semi-stable size distribution (Fig. 3E). Western blotting was conducted to detect exosomal surface markers, such as Alix, TSG101, CD9, CD63 and CD81 (Fig. 3F). Dil-labeled exosomes were shown to be taken up by microglia (Fig. 3G). These findings confirm the successful isolation of exosomes from INCM.

**Fig. 4:**
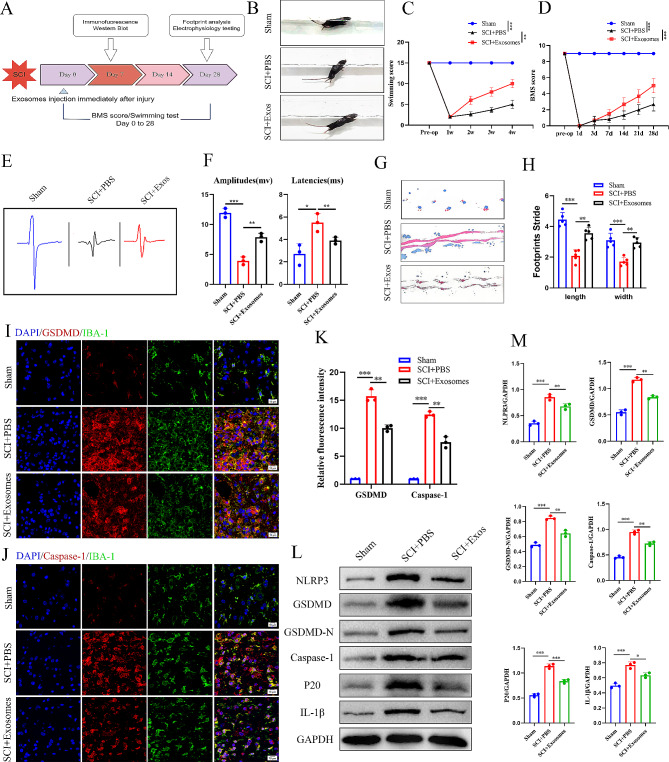
iPSC-NSCs-derived exosomes supress microglia pyroptosis and promote motor function recovery after SCI in vivo. **(A)** Schematic diagram of experimental design; **(B-C)** Typical diagrams of swimming tests in sham group, SCI + PBS and SCI + Exosomes mice at day 28 after injury; **(D)** BMS scores of sham group, treated with PBS and Exosomes mice during the recovery period 28 days after injury; **(E-F)** Electrophysiological assessment with MEP analysis in sham group, SCI + PBS, and SCI + Exosomes mice at day 28 post-injury; **(G-H)** Typical diagrams of footprint tests in sham group, SCI + PBS, and SCI + Exosomes mice at day 28 after injury (*n* = 5 per group); **(I-J)** Representative immunofluorescence staining images of GSDMD/Caspase-1 (red) and IBA-1 (green) in sham group and SCI areas at day 7 after injury in SCI + PBS and SCI + Exosomes mice; **(K)** Quantification of fuorescence intensity of GSDMD and Caspase-1; **(L)** Western blot analysis of pyroptosis-related protein expression at sham group and day 7 after injury in SCI + PBS and SCI + Exosomes mice; **(M)** Quantitative analysis of pyroptosis-related protein. *P < 0.05; **P < 0.01; ***P < 0.001 (*n* = 3 per group)

After immediately injecting exosomes into the injury site, behavioral assessments were conducted at designated time intervals post-SCI (Fig. 4A). Before conducting in vivo experiments, we first tracked the distribution and localization of exosomes in the SCI microenvironment, and the exosomes labeled with Dil red fluorescence were engulfed by microglia/macrophages to ensure that the exosomes could play a regulatory role on them (Figs. S1C-D). Subsequently, we investigated the effects of different concentrations of exosomes on the expression of inflammatory factors after pyroptosis, and found that 20ug/ml was the most effective injection concentration. Moreover, qRT-PCR results showed that compared with mice treated with non-specific exosomes (293T-derived exosomes, Exos_293_), The expression of IL-1β and IL-18 in the spinal cord of iPSC-NSC-Exos mice was significantly reduced, which determined the specificity of the effect observed by iPSC-NSCs (Figs. S1E).

Notably, we observed enhanced recovery in the swimming tests of the iPSC-NSCs-Exos mice. Furthermore, congruent results were obtained from BMS scores and footprint analysis, and electrophysiologic tests revealed more favorable outcomes in mice within the iPSC-NSC-Exos mice (Fig. 4B-H). Compared with the PBS mice, immunofluorescence staining demonstrated a decrease in the number of IBA1^+^/GSDMD^+^ and IBA1^+^/Caspase-1^+^ cells, along with reduced fluorescence intensity in the iPSC-NSCs-Exos mice (Fig. 4I-K). Additionally, western blotting revealed significantly reduced expression levels of NLRP3, GSDMD, GSDMD-N, Caspase-1, p20, and IL-1β in the iPSC-NSCs-Exos mice (Fig. 4L-M), consistent with the immunofluorescence results.

These findings suggest that the iPSC-NSCs-Exos group demonstrates greater efficacy in mitigating microglial/macrophage pyroptosis than the PBS group, possibly attributable to the smaller particle size and enhanced membrane permeability of exosomes. These characteristics may facilitate exosome passage through the blood-spinal cord barrier, thereby exerting a superior inhibitory effect on microglial/macrophage pyroptosis.

### Let-7b-5p is abundantly expressed in iPSC-NSCs-Exos and can be delivered to the microglia after SCI

**Fig. 5:**
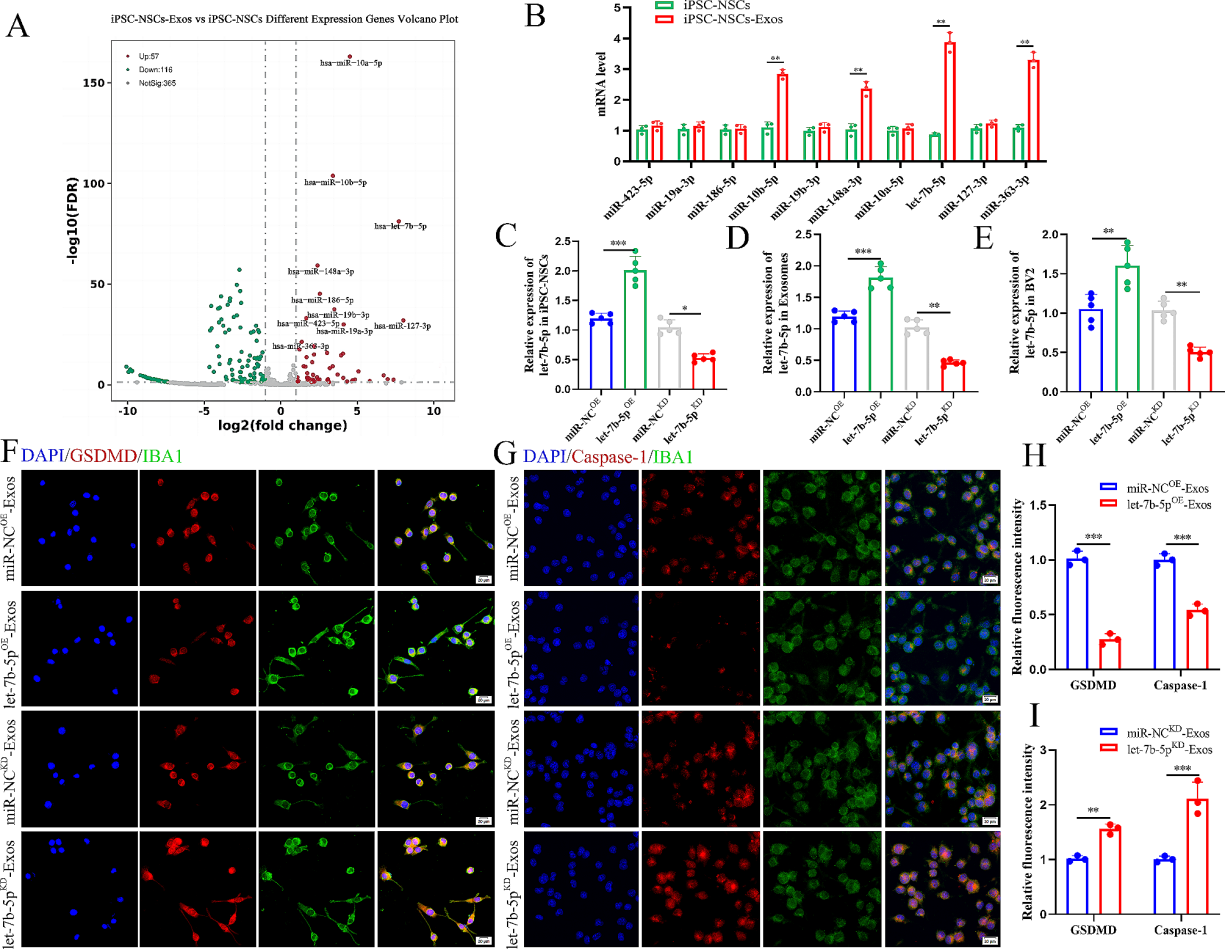
iPSC-NSCs-Exos suppress pyroptosis in BV2 cells by delivering let-7b-5p in vitro. **(A)** Volcano plot of differential expression miRNAs in iPSC-NSCs-Exos compared with iPSC-NSCs; **(B)** qRT-PCR quantitative study of the top 10 significantly differential expression miRNAs (Log2FC > 1) between iPSC-NSCs-Exos and iPSC-NSCs; **(C)**Transfection efciency of let-7b-5p overexpression and knockdown in iPSC-NSCs (*n* = 5 per group); **(D)** The relative expression of let-7b-5p in exosomes derived from iPSC-NSCs in indicated groups (*n* = 5 per group); **(E)** The relative expression of let-7b-5p in BV2 cells administered with miR-NC^OE^-Exos, let-7b-5p^OE^-Exos, miR-NC^KD^-Exos and let-7b-5p^KD^-Exos (*n* = 5 per group); **(F-G)** Representative immunofluorescence staining images of GSDMD/Caspase-1 (red) and IBA-1 (green) in indicated groups; **(H-I)** Quantifcation of fuorescence of GSDMD and Caspase-1 intensity in indicated groups. *P < 0.05; **P < 0.01; ***P < 0.001 (*n* = 3 per group)

Exosomes are composed of various components, including miRNAs, mRNA, and proteins, and are transferred to target cells through intercellular contact. Exosome-loaded miRNAs are essential for coordinating a number of biological processes. In our examination, advanced miRNA sequencing techniques were employed to explore the potential biological mechanisms influencing the impact of iPSC-NSCs-Exos on microglial/macrophage pyroptosis (GSE267739), resulting in the identification of 10 differentially expressed miRNAs (Fig. 5A). Subsequent validation using qRT-PCR confirmed the significant upregulation of 10 miRNAs, including miR-10b-5p, let-7b-5p, miR-148a-3p, and miR-363-3p, with notably elevated expression in iPSC-NSCs-Exos (Fig. 5B). Remarkably, let-7b-5p exhibited the most discernible difference in expression and was consequently chosen as the focal molecule for subsequent investigations.

### Exosomes suppressed microglial/macrophage pyroptosis and motor function recovery post-SCI by delivering let-7b-5p

Based on the aforementioned results, we found that iPSC-NSCs-derived exosomal let-7b-5p can be transferred to microglia. Consequently, we speculated that iPSC-NSCs-Exos regulate post-SCI microglial/macrophage pyroptosis and motor function recovery because let-7b-5p is a biological messenger between iPSC-NSCs and microglia/macrophages.

In order to investigate the effects of exosomal let-7b-5p on the control of microglial/macrophage pyroptosis post-SCI, we used a lentiviral-based method to establish iPSC-NSCs with let-7b-5p overexpression and knockdown, along with their respective negative control groups, designated as let-7b-5p^OE^, miR-NC^OE^, let-7b-5p^KD^ and miR-NC^KD^. The efficiency of transfection was evaluated through qRT-PCR (Fig. 5C). Exosomes were isolated from each of the four groups: let-7b-5p^OE^-Exos, miR-NC^OE^-Exos, let-7b-5p^KD^-Exos and miR-NC^KD^-Exos. We observed significantly higher expression of let-7b-5p in the let-7b-5p^OE^-Exos group compared with the miR-NC^OE^-Exos group, whereas let-7b-5p expression in the let-7b-5p^KD^-Exos group was markedly lower than in the miR-NC^KD^-Exos group (Fig. 5D). Furthermore, the expression level of let-7b-5p in target BV2 microglia was consistent with the trends observed in the exosomes from each group (Fig. 5E). We established an in vitro pyroptosis model using LPS + ATP-treated BV2 cells. In the let-7b-5p^OE^-Exos group, the fluorescence intensity of GSDMD and Caspase-1 was significantly lower compared to the miR-NC^OE^-Exos group, while the opposite effect was observed in the let-7b-5p^KD^-Exos group (Fig. 5F-I).

**Fig. 6:**
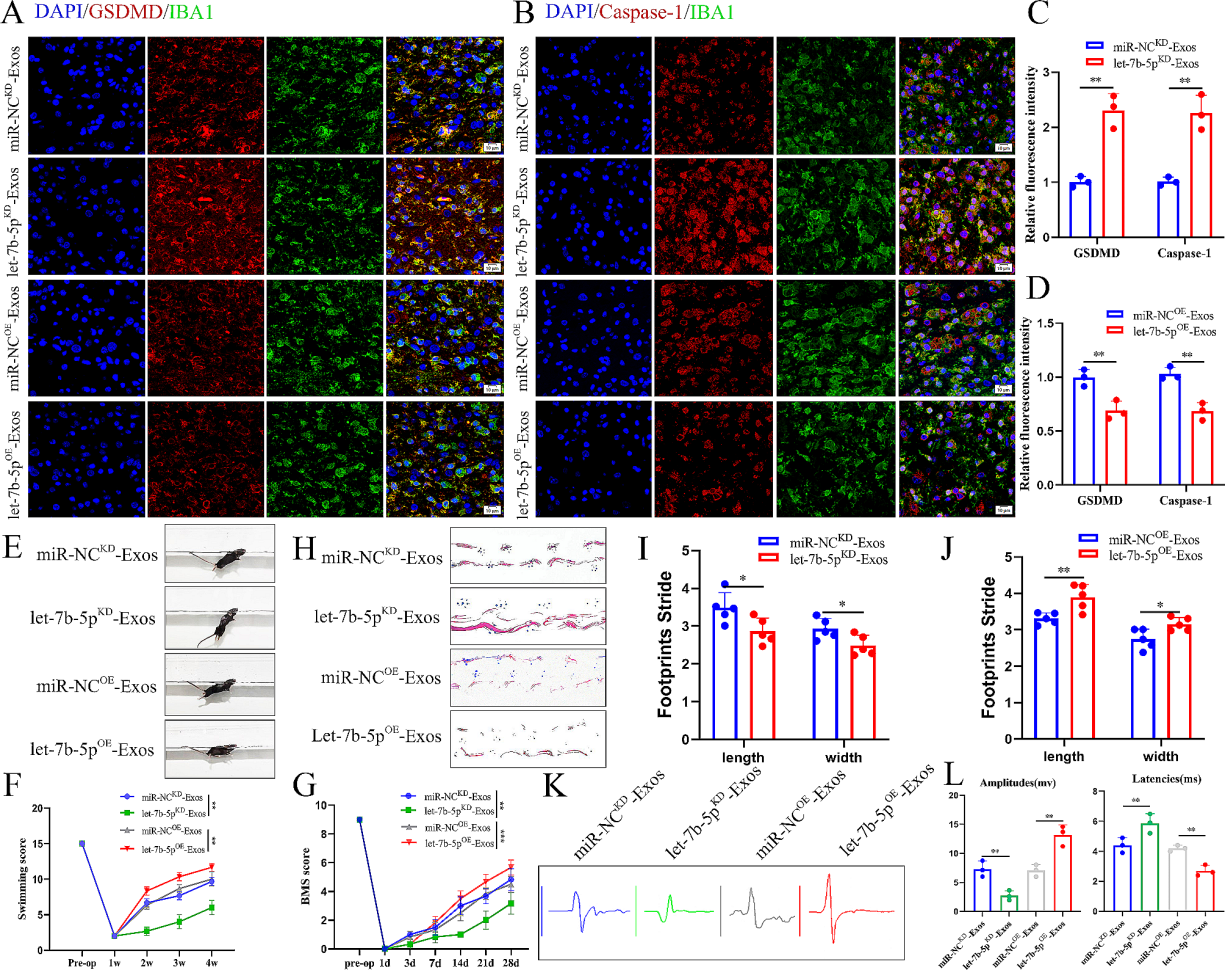
iPSC-NSCs-Exos suppress microglia pyroptosis and promote motor function recovery by delivering let-7b-5p after SCI. **(A-B)** Representative immunofluorescence staining images of GSDMD/Caspase-1 (red) and IBA-1 (green) in indicated groups; **(C-D)** Quantification of fuorescence of GSDMD and Caspase-1 intensity in indicated groups; **(E-F)** Typical diagrams of swimming tests in indicated group mice at day 28 after injury; **(G)** BMS scores of indicated group mice during the recovery period 28 days after injury; **(H-J)** Typical diagrams of footprint tests in indicated group mice at day 28 after injury (*n* = 5 per group); **(K-L)** Electrophysiological assessment with MEP analysis in indicated group mice at day 28 after injury; *P < 0.05; **P < 0.01; ***P < 0.001 (*n* = 6 per group)

After confirming the cellular level effects of let-7b-5p, we elucidated the in vivo function of let-7b-5p in SCI mice. We injected let-7b-5p^OE^-Exos, let-7b-5p^KD^-Exos, miR-NC^OE^-Exos, and miR-NC^KD^-Exos into WT mice post-SCI. Immunofluorescence staining showed that GSDMD and Caspase-1 fluorescence intensity was significantly lower in the let-7b-5p^OE^-Exos group compared to the miR-NC^OE^-Exos group. Conversely, the let-7b-5p^KD^-Exos group exhibited a significant increase in GSDMD and Caspase-1 fluorescence intensity compared to the miR-NC^KD^-Exos group (Fig. 6A-D). Behavioral analysis experiments were conducted to observe the enhancement in motor function of SCI mice at designated time intervals. Analysis based on swimming tests (Fig. 6E-F), BMS scores (Fig. 6G), footprint analysis (Fig. 6H-J), and electrophysiology tests (Fig. 6K-L) revealed that reduced let-7b-5p expression was detrimental to motor function recovery, while elevated let-7b-5p expression enhanced motor function.

**Fig. 7:**
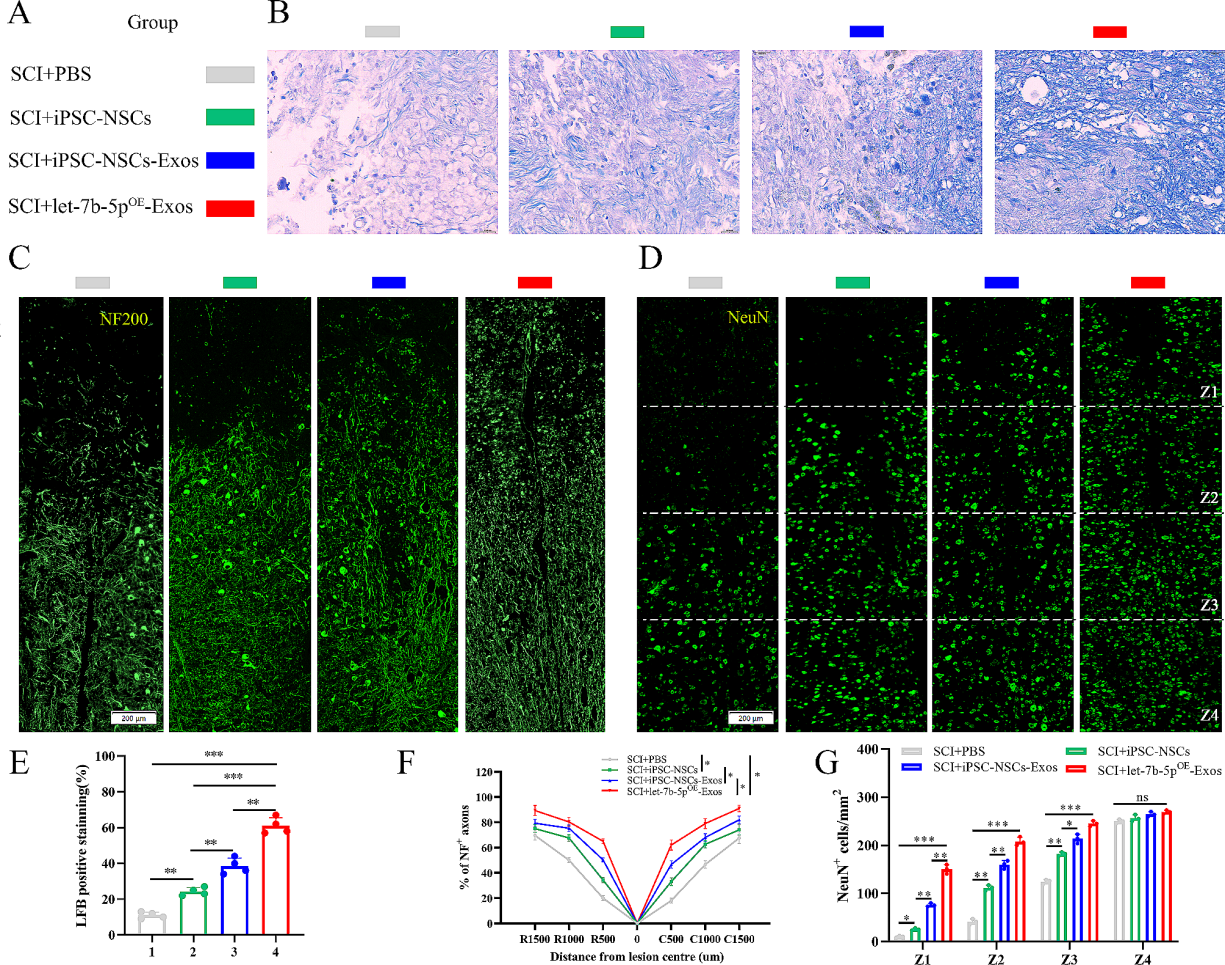
let-7b-5p^OE^-Exos promotes remyelination and axonal regeneration necessary for locomotion. **(A)** Detailed information on the experimental group in this part of the experiment; **(B)** Representative images of LFB staining of myelin sheaths in the indicated groups 28 days after injury; **(C)** Representative immunofluorescence images of NF200^+^ in injured spinal cords at day 28 after injury; **(D)** Representative immunofluorescence images of NeuN^+^ neurons in the Z1-Z4 region adjacent to the lesion core at day 28 after injury; **(E)** Quantification of LFB positive areas of the spinal cord (*n* = 4 per group); **(F)** Quantitative analysis of the percentage of NF200^+^ areas at the indicated distances from the center of SCI lesions to the total area of distant uninjured axons; **(G)** Quantification of NeuN^+^ neurons in the Z1-Z4 region adjacent to the lesion core at day 28 after injury. *P < 0.05; **P < 0.01; ***P < 0.001 (*n* = 3 per group)

We conducted additional analyses to test how let-7b-5p^OE^-Exos produced this promising effect. As is well-known, remyelination and axon regeneration are essential for the recovery of motor function. Consequently, at 28 days post-SCI, we used LFB staining to evaluate the degree of axonal myelination at the lesion site. The detailed information regarding the experimental group is presented in (Fig. 7A). Compared with the PBS group, the residual amount of myelin in the iPSC-NSCs, iPSC-NSCs-Exos and let-7b-5p^OE^-Exos groups was significantly higher, and the let-7b-5p^OE^-Exos groups treatment effect was best among these groups (Fig. 7B and E). We then proceeded to use Neurofilament-200 (NF200) to evaluate axon density or status in the lesioned core of the injured spinal cord at 28 days post-SCI. In the PBS group, few NF200^+^ axons were observed at the injury site, while in the iPSC-NSCs, iPSC-NSCs-Exos and let-7b-5p^OE^-Exos treatment groups, more NF200^+^ labeled fibers traversed the glial scar, indicating axonal extension. Among these treatment groups, the let-7b-5p^OE^-Exos group exhibited the most extended neurofilaments compared to the other groups (Fig. 7C and F). To further substantiate the aforementioned findings, we labeled living neurons in specific locations (Z1-Z4) using the neuronal marker NeuN within the injured spinal cords of the four groups. This region was situated at a defined distance from the lesion boundary, as detailed in previous studies [29]. Consistent with the results of NF200 fluorescence staining, the count of viable NeuN^+^ neurons in the Z1-Z3 region of the iPSC-NSCs, iPSC-NSCs-Exos, and let-7b-5p^OE^-Exos treatment groups was markedly greater than that in the PBS group. Notably, the let-7b-5p^OE^-Exos treatment group displayed the highest number of surviving NeuN^+^ neurons (Fig. 7D and G). In summary, our findings suggest that iPSC-NSCs-Exos inhibits microglial/macrophage pyroptosis and alleviates myelin sheath destruction, and axon outgrowth impairment by delivering let-7b-5p, thereby promoting the recovery of motor function post-SCI.

### Let-7b-5p negatively regulates LRIG3

**Fig. 8:**
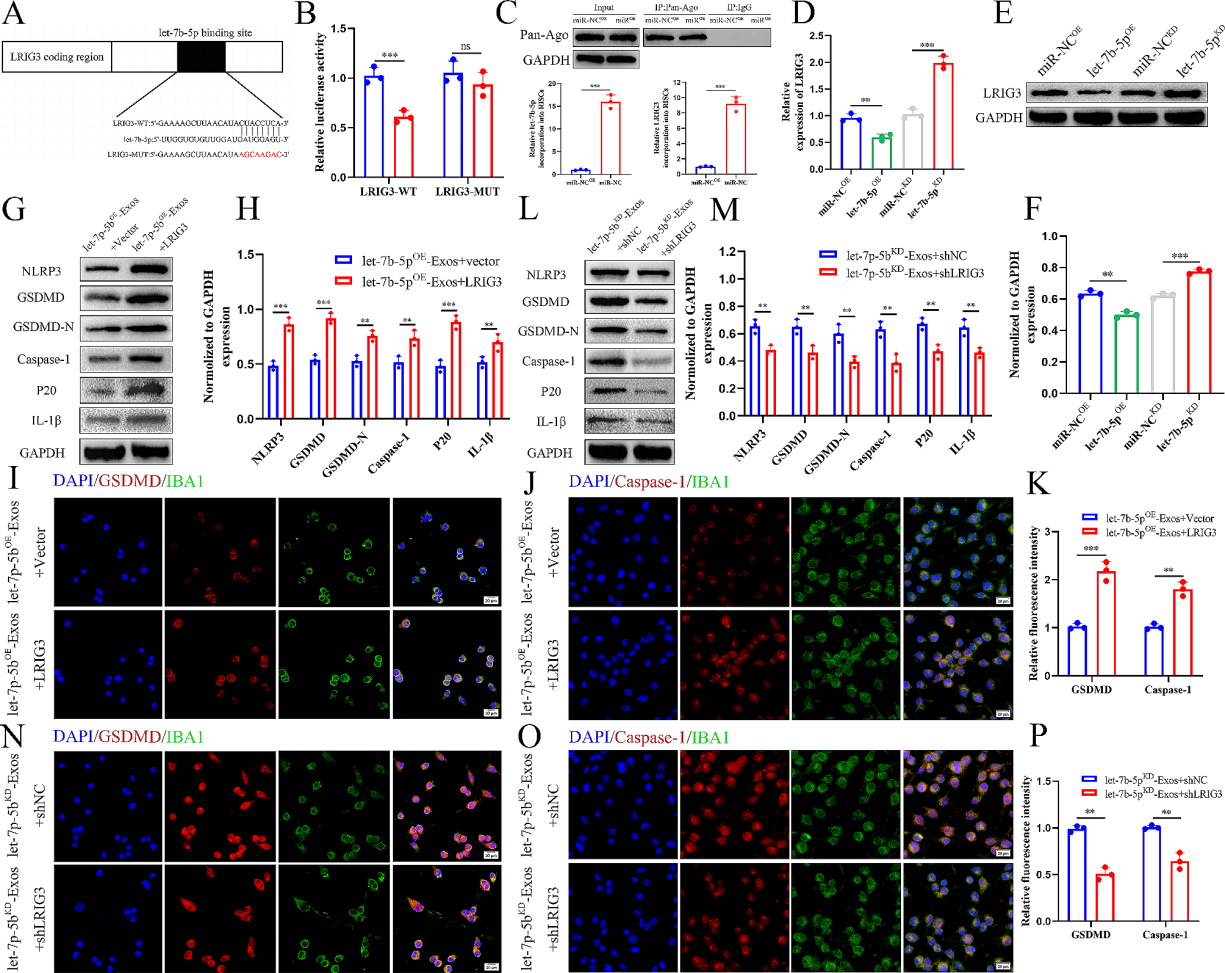
Exosomal let-7b-5p suppress microglia pyroptosis by regulating LRIG3 expression. **(A)** Exosomal let-7b-5p regulates LRIG3 by directly targeting the 3′-UTR; **(B)** Luciferase report assay was performed to confirm LRIG3 is the target gene of let-7b-5p; **(C)** Immunoprecipitation of the Ago2/RISC (RNA-induced silencing complex) using the Pan-Ago2 antibody in BV2 microglia overexpressing miR-NC or let-7b-5p. IgG was used as a negative control and GAPDH was used as an internal control; **(D)** The mRNA level of LRIG3 in BV2 cells after treatment with let-7b-5p^OE^-Exos and let-7b-5p^KD^-Exos; **(E)** The protein level of LRIG3 in BV2 cells after treatment with let-7b-5p^OE^-Exos and let-7b-5p^KD^-Exos; **(F)** Quantitative analysis of LRIG3 protein; **(G-J)** Rescue experiments for let-7b-5p inhibition were conducted by downregulating LRIG3 in microglia. Microglia pyroptosis was detected by western blot analysis and immunofuorescence; **(K-N)** Rescue experiments for let-7b-5p overexpression were carried out by the ectopic expression of LRIG3 in microglia. Microglia pyroptosis was detected by western blot analysis and immunofuorescence. *P < 0.05; **P < 0.01; ***P < 0.001 (*n* = 3 per group)

To further elucidate the mechanism of exosomal let-7b-5p, we used online databases of mRNA targets to analyze and predict potential mRNA target genes of let-7b-5p. LRIG3—one of the potential mRNA target genes of let-7b-5p—is linked to inflammation, and we speculated that let-7b-5p recognizes and binds to its 3’ untranslated region (3’-UTR). To validate our speculation, we constructed both WT and MUT 3’-UTR sequences of LRIG3 based on the potential binding site of LRIG3 (Fig. 8A) and co-transfected them with let-7b-5p sequences into 293T cells. The luciferase reporter analysis indicated a substantial drop in luciferase activity in the WT-LRIG3-3’UTR and let-7b-5p^OE^ co-transfected group compared with the WT-LRIG3-3’UTR and miR-NC^OE^ group. In contrast, luciferase activity showed no discernible difference between the control group and the co-transfected group with let-7b-5p^OE^ and MUT-LRIG3-3’UTR (Fig. 8B), indicating that LRIG3’s 3’ UTR can specifically bind to let-7b-5p. Following let-7b-5p overexpression, RNA-ChIP analysis was employed to precisely quantify LRIG3 mRNA within the Ago2/RNA-induced silencing complex (RISC). Cells overexpressing let-7b-5p exhibited an increased incorporation of LRIG3 into the RISC (Fig. 8C). Furthermore, we observed that LRIG3 mRNA and protein expression levels decreased in response to overexpressing let-7b-5p, whereas LRIG3 mRNA and protein expression levels increased in response to let-7b-5p knockdown (Fig. 8D-F). This provides further confirmation that LRIG3 is a downstream target gene of let-7b-5p.

### Let-7b-5p affects microglial/macrophage pyroptosis by targeting LRIG3

To further investigate the relationship between exosomal let-7b-5p and LRIG3, a series of in vitro rescue experiments were conducted. Firstly, we overexpressed LRIG3 in BV2 cells through lentiviral transduction and co-treated them with let-7b-5p^OE^-Exos. Western blotting analysis revealed that LRIG3 overexpression increased the activation of microglial pyroptosis (Fig. 8G-H), a finding further confirmed through immunofluorescence staining (Fig. 8I-K). Additionally, we employed shRNA to suppress LRIG3 expression in BV2 cells and co-treated them with let-7b-5p^KD^-Exos. Western blotting analysis indicated that LRIG3 inhibition reduced the activation of microglial pyroptosis (Fig. 8L-M), with immunofluorescence staining providing further confirmation (Fig. 8N-P).

## Discussion

SCI is a severe condition that substantially impairs the life quality of patients, and its treatment has been a longstanding challenge in the medicinal field. Among the factors contributing to poor outcomes in SCI, SCI-induced neuronal damage and inflammatory responses play a crucial role [30]. Pyroptosis—a novel form of inflammation-related cell death—has been studied in various diseases [31]. However, its potential mechanisms in microglial/macrophages post-SCI remain unclear. Our study revealed a significant increase in pyroptosis within microglial/macrophages post-SCI, consistent with our previous study [14]. These results suggest that pyroptosis may be pivotal in neuroinflammatory responses and secondary injuries.

The transplantation of cells offers a range of therapeutic possibilities, with NSCs being particularly significant in SCI repair because of their numerous advantages and applications [32]. Christina Brown and colleagues have demonstrated that NSCs exert anti-inflammatory and immune-regulatory effects, effectively mitigating excessive inflammatory responses at the injury site and consequently diminishing secondary damage [33]. Myriam Cayre discovered the participation of NSCs in remyelination and axon regeneration, thereby creating conducive conditions to regenerate impaired nerve fibers [34]. Additionally, Elizabeth D. Kirby and colleagues found that the secretion of trophic factors by NSCs plays a significant role in safeguarding and facilitating the survival and growth of damaged neurons [35]. Furthermore, Anderson AJ revealed substantial enhancements in motor function after the transplantation of NSCs in animal SCI models. This finding implies that NSCs not only exhibit viability and differentiation within the host organism but also exert an influence on the injury microenvironment to promote the recovery of neural function [36]. Nevertheless, it is important to acknowledge that NSCs used in the repair of SCI possess certain noteworthy constraints.

Erin Lavik and his colleagues demonstrated that NSCs can differentiate into glial cells, thereby promoting the formation of glial scars and impeding neural regeneration [37]. Consequently, combining NSCs transplantation with other cells in the SCI site is often necessary to facilitate synergistic effects. Furthermore, the availability of NSCs is constrained, mainly because of ethical and legal limitations on the utilization of NSCs derived from human embryonic tissues in clinical settings [18]. Additionally, the successful survival and integration of transplanted NSCs at the injury site pose significant challenges, influenced by individual variations and potential interactions with the host’s immune system [38].

The advent of iPSC technology has yielded notable advancements in cell transplantation therapy within clinical settings [39]. iPSCs present a distinct advantage by bypassing ethical constraints and mitigating concerns related to immune rejection, thereby paving the way for novel prospects in the medical domain [40]. Studies have demonstrated substantial therapeutic efficacy of iPSCs in various afflictions, including but not limited to hematological disorders, endocrine dysfunctions, and cardiovascular pathologies [41–43]. The redirection of iPSCs towards neural stem cells (iPSC-NSCs) lineage holds promise for treating neurological disorders, including multiple sclerosis and Parkinson’s disease [44, 45]. Moreover, iPSC-NSCs demonstrate advantageous characteristics conducive to their differentiation into neurons within SCI models. Nevertheless, the exploration of their potential contribution to ameliorating microglial/macrophage pyroptosis post-SCI remains inadequately investigated. In our study, we transplanted iPSC-NSCs into SCI mice and observed a beneficial effect of iPSC-NSCs in mitigating microglial/macrophage pyroptosis, preserving myelin integrity, and promoting axon growth, ultimately contributing to the functional recovery of SCI mice.

However, it is imperative to acknowledge the potential risks associated with iPSC transplantation. Nissim Benvenisty et al. reported that the pluripotency of iPSCs may give rise to tumorigenicity [46]. This concern arises from the possibility of retroviruses used during the reprogramming procedure, randomly integrating into the host genome. Such integration can disrupt cell cycle regulation, potentially activating oncogenes or inhibiting tumor suppressor genes. In conclusion, when iPSC technology and its derivative iPSC-NSCs exhibit immense potential in the medical field, their clinical implementation necessitates comprehensive safety assessments and validation [47]. In subsequent studies, researchers must focus on the biological attributes of these cells to optimize therapeutic effectiveness and mitigate potential hazards. Such endeavors will facilitate pioneering progress in cell therapy, presenting inventive remedies for various ailments. Some studies have explored the generation of enriched exosomes through in vitro-cultured iPSCs to mitigate the potential adverse effects of iPSC transplantation [48, 49]. Exosomes are nanosized vesicles that play a pivotal role in intercellular communication by transporting genetic materials, such as proteins and functional RNA, thus facilitating cellular signaling [50]. Fawad Ali and others confirmed that tail vein injection of iPSC-derived exosomes (iPSC-Exos) effectively improves motor function in SCI mice and regulates the expression of inflammatory factors associated with this process [51]. However, our understanding of the potential role of iPSC-NSCs-Exos in treating SCI has remained limited. To comprehensively examine the therapeutic potential of iPSC-NSCs-Exos in the context of SCI, we transplantated iPSC-NSCs-Exos into SCI mice. Our findings support the effectiveness of iPSC-NSCs-Exos in ameliorating microglial/macrophage pyroptosis, preserving the integrity of myelin, and fostering axon growth. Ultimately, these effects contribute to the restoration of functionality in SCI mice. Exosomes serve as agents of intercellular communication and possess substantial biological significance in treating SCI [52]. Nonetheless, additional investigations to elucidate the exact effects of iPSC-NSCs-Exos in SCI therapy may encompass examining exosomes composition and their potential involvement in molecular mechanisms. In this context, we used miRNA microarray assay to discern the higher abundance of let-7b-5p in iPSC-NSCs-Exos compared with iPSC-NSCs. We also observed the successful transfer of let-7b-5p to microglia after exosomes treatment. The critical function of let-7b-5p in inhibiting inflammatory responses has been brought to light by recent investigations [53, 54]. The enrichment of let-7b-5p within exosomes potentially assumes a pivotal role in SCI, as let-7b-5p suppresses the occurrence of microglial/macrophage pyroptosis and promotes functional recovery. We speculated that exosomes serve as vehicles for miRNAs, facilitating the transfer of biologically active let-7b-5p to microglia/macrophages. Nevertheless, additional genes may possess therapeutic properties autonomously or when combined with exosomes. Subsequent investigations will further explore the precise mechanisms underlying the enhancement of functional recuperation in SCI mice through iPSC-NSCs-Exos.

LRIG3—a member of the LRIG gene family (leucine-rich repeats and immunoglobulin-like domains, LRIG)—was discovered in a study of negative feedback regulation in the epidermal growth factor receptor (EGFR) signaling pathway [55]. It plays a crucial role in the pathophysiology of various cancers. Current research has confirmed that downregulating LRIG3 in an Alzheimer’s disease rat model can inhibit oxidative stress damage and inflammatory injury by modulating the PI3/Akt pathway [56]. Notably, our study verified that LRIG3 is a downstream target gene of let-7b-5p and is negatively regulated by let-7b-5p. Subsequent rescue experiments demonstrated that let-7b-5p reduces pyroptosis in microglial/macrophages by inhibiting the expression of LRIG3, explaining how iPSC-NSCs reduce pyroptosis through exosomes.

In summary, the present study represents the first confirmation that iPSC-NSCs and their exosomes effectively suppress pyroptosis and neuroinflammation in microglial/macrophages subjected to SCI and LPS stimulation. These interventions alleviate the formation of glial scars, maintain the integrity of myelin, and facilitate the growth of axons, ultimately restoring functional abilities in SCI mice. The nanoscale exosomes demonstrate the ability to cross the blood-spinal cord barrier, facilitating the administration of therapeutic exosomal miRNAs to the SCI region and offering great potential for clinical utilization. Therefore, iPSC-NSCs and their exosomes, in conjunction with miRNAs, could emerge as innovative tools to treat SCI.

## Conclusion

Our research unveils a potential mechanism wherein exosomes derived from iPSC-NSCs shuttle let-7b-5p to facilitate the recovery of motor function after SCI. Enriched levels of exosomal let-7b-5p mitigate pyroptosis in microglia/macrophages, thereby reducing the secondary inflammatory response following SCI and enhancing therapeutic potential by suppressing its target gene LRIG3. The combined treatment of iPSC-NSCs-Exos and miRNAs holds promising prospects as an innovative therapeutic approach for SCI. The communication and regulation between cells and the pathological mechanism of spinal cord injury is an extremely complex network. This study only involves one of the pathways, which has certain limitations. We will conduct further research in the subsequent experiments.

### Electronic supplementary material

Below is the link to the electronic supplementary material.

Supplementary Material 1

## Data Availability

No datasets were generated or analysed during the current study.

## Abbreviations

SCI: spinal cord injury
Exos: Exosomes
SPF: Specific Pathogen Free
TEM: Transmission electron microscopy
NTA: Nanoparticle tracking analysis
BMS: Basso mouse scale
MEPs: Motor-evoked potentials
NSCs: Neural stem cells
iPSCs: Induced pluripotent stem cells
iPSC-NSCs: Induced pluripotent stem cell-derived neural stem cells
NF200: Neurofilament-200
EGFR: Epidermal growth factor receptor
LPS: Lipopolysaccharides
ATP: Adenosine triphosphate
