# Supplementary figures and images for: IPSC-NSCs-derived exosomal let-7b-5p improves motor function after spinal cord Injury by modulating microglial/macrophage pyroptosis
Source: J Nanobiotechnology. 2024 Jul 9;22:403. doi: 10.1186/s12951-024-02697-w (PMC11232148; doi:10.1186/s12951-024-02697-w)

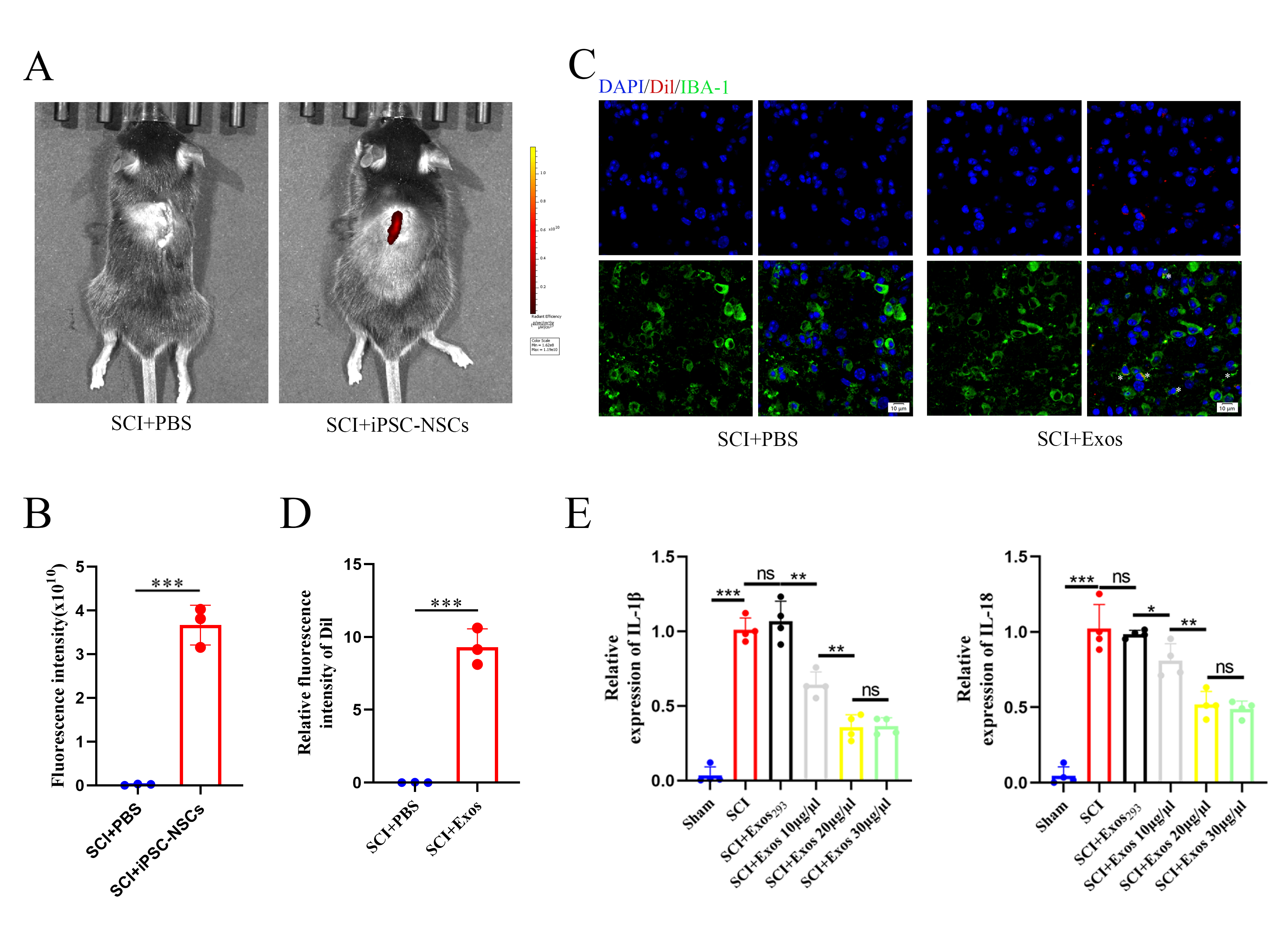

Supplement: Supplementary file 1 — Supplementary Material 1 [file 12951_2024_2697_MOESM1_ESM.tif]
